# Preparation and Characterization of Soy Isoflavones Nanoparticles Using Polymerized Goat Milk Whey Protein as Wall Material

**DOI:** 10.3390/foods9091198

**Published:** 2020-08-31

**Authors:** Mu Tian, Cuina Wang, Jianjun Cheng, Hao Wang, Shilong Jiang, Mingruo Guo

**Affiliations:** 1 Key Laboratory of Dairy Science, Northeast Agricultural University, Harbin 150030, China; tm18646175069@163.com (M.T.); wangcuina@jlu.edu.cn (C.W.); cheng577@163.com (J.C.); jlu_wh@126.com (H.W.); 2 HeiLongJiang FeiHe Dairy Co., Ltd., Beijing 100015, China; jiangshilong@feihe.com; 3 Department of Nutrition and Food Sciences, College of Agriculture and Life Sciences, University of Vermont, Burlington, VT 05405, USA

**Keywords:** polymerized goat milk whey protein, soy isoflavones, nanoparticle, physicochemical property

## Abstract

Soy isoflavones (SIF) are a group of polyphenolic compounds with health benefits. However, application of SIF in functional foods is limited due to its poor aqueous solubility. SIF nanoparticles with different concentrations were prepared using polymerized goat milk whey protein (PGWP) as wall material. The goat milk whey protein was prepared from raw milk by membrane processing technology. The encapsulation efficiencies of all the nanoparticles were found to be greater than 70%. The nanoparticles showed larger particle size and lower zeta potential compared with the PGWP. Fourier Transform Infrared Spectroscopy indicated that the secondary structure of goat milk whey protein was changed after interacting with SIF, with transformation of α-helix and β-sheet to disordered structures. Fluorescence data indicated that interactions between SIF and PGWP decreased the fluorescence intensity. All nanoparticles had spherical microstructure revealed by Transmission Electron Microscope. Data indicated that PGWP may be a good carrier material for the delivery of SIF to improve its applications in functional foods.

## 1. Introduction

Soy isoflavones (SIF), as bioactive substances, are the main secondary metabolites in soybeans. SIF have been found to possess several potential health benefits such as anticancer [1], reducing menopausal syndrome [2], and preventing osteoporosis [3]. Because of their health benefits, SIF have been recommended as a functional ingredient for formulation of healthy foods and pharmaceutical products. However, due to its low solubility in water, poor bioavailability, and high susceptibility to degradation under oxygen, light, and heating conditions [4], the application of SIF in the food industry is limited. Several approaches have been developed to solve these problems. Functional SIF nanoparticles were prepared by antisolvent precipitation method to improve the water dissolution rate [4]. Wang et al. [5] reported that SIF were microencapsulated in gel beads of soybean hull polysaccharides.

Goat milk whey protein has attracted increasing interest in recent years. The major components in goat milk whey protein are β-lactoglobulin (β-LG) and α-lactalbumin (α-LA), which have excellent emulsifying and foaming properties [6,7]. In recent years, membrane processing technology has been widely used in the dairy industry in an industrial scale due to its low energy consumption, low temperatures, and reduction of environmental contaminants. The goat milk whey protein was prepared from clarified cheese whey by microfiltration (MF) and ultrafiltration (UF) technology, which has good gelation and emulsifying properties [8]. The emulsifying property of goat milk whey protein can be improved by heat treatments [9]. During heating, the polypeptide chains of protein unfolded, and the sulfhydryl groups were exposed to form polymerized whey proteins (PWP) which can be used as an encapsulating material with improved functional properties [10,11]. Because of the protein surface-active property, PWP has a strong affinity towards different ligands [12]. Information about the application of polymerized goat milk whey protein (PGWP) as a bioactive compound carrier is very limited.

Therefore, the aim of this work was to prepare SIF nanoparticles using PGWP as wall material. The PGWP was prepared directly from goat milk by membrane processing technology and the physicochemical properties of nanoparticles were characterized.

## 2. Materials and Methods

### 2.1. Materials

Raw goat milk (≥8.15% nonfat solids, 3.86% protein, and 4.02% fat, *w*/*v*) was purchased from a local market (Feihe Dairy Industry Co., Ltd., Harbin, China). Soy isoflavones (SIF, ≥80% purity) were provided by Beijing Solarbio Science and Technology Co., Ltd. (Beijing, China). Deionized water was prepared by a water filtration device (Millipore Corp., Bedford, MA, USA).

### 2.2. Nanoparticle Preparation

#### 2.2.1. Preparation of Goat Milk Whey Protein Concentrate

Raw goat milk was heated to 55 °C and skimmed with a separator (SA 10-T, Frautech SRL, Thiene, Italy) to obtain skimmed goat milk and cream. The skimmed goat milk was filtered by microfiltration (MF, 0.1 μm, 50 °C). MF permeate was ultrafiltrated (UF) by a cut-off 10 kDa spiral-wound membrane to 10-fold, and the UF retentate was electrodialyzed (ED) to remove 85% of salt [13]. Subsequently, the concentrated goat milk whey protein was freeze dried in a freeze dryer (Alpha 1-2, Marin Christ Inc., Osterode, Germany) to obtain goat milk whey protein powder (80.99% protein, 18.67% lactose, and 0.34% ash, *w*/*w*).

#### 2.2.2. Preparation of Polymerized Goat Milk Whey Protein

The goat milk whey protein powder was dissolved in deionized water to obtain a 10% (*w*/*v*) goat milk whey protein solution and stored at 4 °C for 12 h to complete hydration. The solution was adjusted to pH 7.7 with 1 M sodium hydroxide, heated to 75 °C for 25 min with continuous stirring, and was then quickly cooled to room temperature and marked as polymerized goat milk whey protein (PGWP).

#### 2.2.3. Preparation of Soy Isoflavones Solution

The soy isoflavones (SIF) were dissolved in 70% ethanol (5 mg/mL) by stirring using a magnetic stirrer (IKA, Staufen, Germany), and then the solution was heated to 50 °C for 1 h to obtain a clear solution. The container was wrapped with aluminum foil.

#### 2.2.4. PGWP-SIF Nanoparticle Preparation

The PGWP-SIF solutions were prepared by combining PGWP solution, SIF solution, and deionized water to get different concentrations of SIF (2.1, 2.4, 2.7, and 3.0 mg/mL), while the content of PGWP remained at 40 mg/mL. All samples were stirred for 2 h to obtain a stable system. The mixed solution was treated to remove ethanol by nitrogen gas, and deionized water was added to maintain the original volume, and it was stored in darkness. In this study, samples of SIF-loaded PGWP nanoparticles with different SIF concentrations were termed as PGWP-SIF-A (2.1 mg/mL), PGWP-SIF-B (2.4 mg/mL), PGWP-SIF-C (2.7 mg/mL), and PGWP-SIF-D (3.0 mg/mL), respectively. PGWP (40 mg/mL) was set as a control.

### 2.3. Encapsulation Efficiency Determination

The encapsulation efficiency of SIF within PGWP was measured using a previous method with some modifications [14]. The PGWP-SIF solutions were centrifuged at 5500× *g* for 20 min (25 °C), and the concentration of SIF in the supernatant was measured at 261 nm using a UV-visible spectrophotometer (UV-2550, Shimadzu, Tokyo, Japan). The linear regression of absorption versus concentration was conducted, and the regression equation was calculated. The equation is y = 0.1079x − 0.0223, R^2^ = 0.9991. The encapsulation efficiency was calculated as follows in Equation (1):

Encapsulation efficiency (%) = C_0_/C × 100 (1)

where C_0_ is the concentration of SIF in the supernatant after centrifugation, and C is the concentration of SIF in the nanoparticle.

### 2.4. Particle Size and Zeta Potential Analysis

Particle size and zeta potential were carried out using a Malvern Zetasizer Nano ZS90 (Malvern Instruments Ltd., Worcestershire, UK). The PGWP and PGWP-SIF solutions were diluted to a protein concentration of 0.1% with deionized water [15]. The refractive indexes for protein and water were 1.450 and 1.333, respectively. All measurements were performed in triplicate.

### 2.5. Rheological Properties Measurement

Rheological properties of the PGWP and PGWP-SIF solutions were analyzed by a rheometer (Thermo Rheometer, San Jose, CA, USA) equipped with a diameter of 35 mm plate at 25 °C, according to Khan et al. [11]. Flow ramp measurement was carried out using shear rate from 0.1 to 300 s^−1^. For peak hold analysis, apparent viscosity data was recorded by keeping shear rate at 200 s^−1^ for 60 s.

### 2.6. Fourier Transform Infrared (FT-IR) Spectroscopy

Fourier Transform Infrared (FT-IR) spectra of samples were obtained using a FTIR spectrometer (Thermo Electron Scientific Instruments Corporation, San Jose, CA, USA) with a pressurized tablet method. The SIF, PGWP, and PGWP-SIF solutions were pre-frozen at −80 °C for 4 h and freeze dried at 4 °C for 12 h. Solid samples were mixed with potassium bromide (KBr) and ground into fine powder. The wavenumber ranged from 4000 to 400 cm^−1^ with a resolution of 4 cm^−1^ and 32 scans [16]. The spectral region ranges of 1600–1700 cm^−1^ were applied to calculate the secondary structure of protein using Peak FIT software. Bands between 1610–1637 cm^−1^ and 1680–1692 cm^−1^ belong to β-sheet; bands between 1638–1648 cm^−1^ belong to random coil; bands between 1649–1660 cm^−1^ belong to α-helix; and bands between 1660–1680 cm^−1^ belong to β-turn. The band area was calculated using the Gaussian function [17].

### 2.7. Fluorescence Spectroscopy

Fluorescence measurement was performed using an F-7000 fluorescence spectrophotometer (Hitachi Ltd., Tokyo, Japan). The excitation wavelength was set at 280 nm, and the emission was collected from 300 to 500 nm with both slit width at 2.5 nm. The emission spectra were collected at a photomultiplier tube voltage of 500 V with scan rate at 240 nm/min. The PGWP and PGWP-SIF solutions were incubated in water baths at 298, 303, and 308 K for 30 min to achieve equilibrium before measuring [18]. Synchronous fluorescence spectra were recorded at 260–320 nm (Δλ = 15 nm) and 240–320 nm (Δλ = 60 nm) [19].

### 2.8. Differential Scanning Calorimetry (DSC)

Thermal properties of the samples were analyzed using Differential Scanning Calorimetry (Mettler Toledo, DSC 3, Zurich, Switzerland) according to method by Khan et al. [11] with some modifications. All solid samples (about 5 mg) were sealed in aluminum pans and heated from 30 to 300 °C at 10 °C/min under a nitrogen flow rate of 50 mL/min. An empty sealed aluminum pan was used as a control.

### 2.9. Transmission Electron Microscopy (TEM)

Images of samples were obtained using transmission electron microscopy (H-7650, Hitachi High-Technologies, Tokyo, Japan) as described by Ghorbani Gorji et al. [20]. The PGWP and PGWP-SIF solutions were diluted to the appropriate concentration, and 10 μL of the sample was placed on a carbon copper and dyed with a negative staining method. The sample was air dried before imaging.

### 2.10. Statistical Analysis

Data of triplicate experiments were statistically analyzed and expressed as mean ± standard deviation. Analysis of variance (*P* < 0.05) and Tukey’s test were carried out using SPSS 20 software (SPSS Inc., Chicago, IL, USA).

## 3. Results

### 3.1. Encapsulation Efficiency

**Figure 1:**
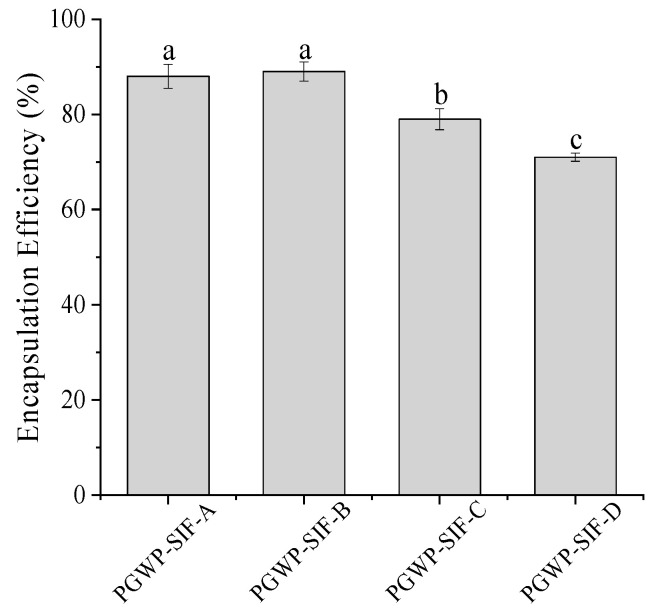
Effect of the soy isoflavone (SIF) contents on encapsulation efficiency of polymerized goat milk whey protein (PGWP)-SIF. Different subscript letters indicate a significant difference (*P* < 0.05). Error bars represent standard deviation of the means.

Encapsulation efficiency of the nanoparticles is shown in Figure 1. The encapsulation efficiencies of all samples were greater than 70%, and the SIF concentration at 2.4 mg/mL had the highest encapsulation efficiency (89%). The encapsulation efficiency decreased as SIF content increased, suggesting that a portion of SIF were not embedded into the polymerized goat milk whey protein matrix at higher SIF concentration. Similar findings were reported by Patel et al. [21] who found that as curcumin concentration increased, encapsulation efficiency of zein-curcumin decreased.

### 3.2. Particle Size and Zeta Potential

**Figure 2:**
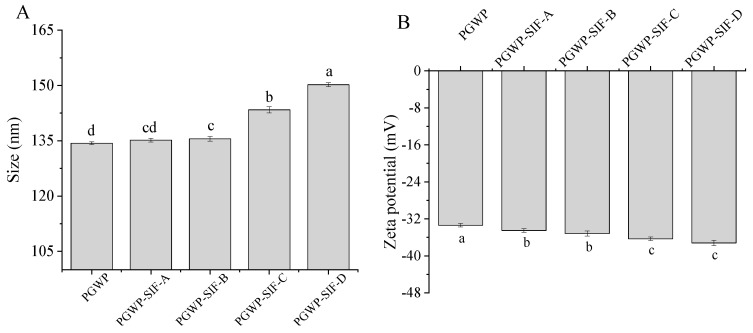
Effects of the SIF contents on particle size (**A**) and zeta potential (**B**) of PGWP-SIF. Different subscript letters indicate a significant difference (*P* < 0.05). Error bars represent standard deviation of the means.

Particle size of PGWP and PGWP-SIF are shown in Figure 2A. All PGWP-SIF samples showed larger particle sizes than that of PGWP. This can be attributed to the fact that the PGWP had more hydrophobic groups, and SIF were entrapped in or absorbed on protein to form compact nanoparticles [22]. The particle size of nanoparticles was dependent on the concentrations of SIF in PGWP-SIF. At low SIF concentrations (at 2.1 and 2.4 mg/mL), there was no significant difference (*P >* 0.05) in particle size, which may be because the majority of SIF were entered into the hydrophobic core of protein. However, at higher SIF concentrations (the SIF concentration at 2.7 and 3.0 mg/mL), a significant (*P* < 0.05) increase in particle size was found, which suggested that more SIF were absorbed at the surface of PGWP until the surface was saturated. The phenomenon was similar to previous results reported by Rodríguez et al. [23], where at low green tea polyphenols contents, the particle size was maintained that of the β-lactoglobulin, and at large green tea polyphenols contents, the particle size was increased with the increasing of green tea polyphenol concentration.

Zeta potential is related to the stability of the systems. Values of zeta potential below –30 mV indicates a stable solution, which may be due to strong electrostatic repulsion [24]. Values of zeta potential in PGWP and PGWP-SIF are shown in Figure 2B. The PGWP-SIF showed lower value of zeta potential than PGWP (*P* < 0.05), suggesting that SIF adhered to PGWP and reduced the negative charge of PGWP. A similar tendency was reported by Von Staszewski et al. [25], who observed that the addition of green tea polyphenols resulted in a decrease in zeta potential values of the β-lactoglobulin-green tea polyphenols complexes. Increasing SIF content from 2.1 mg/mL to 3.0 mg/mL showed an increase of negative charge among PGWP-SIF samples, which indicated that the large number of the embedded SIF entrapped in the core had not affected the surface charge of protein [11].

### 3.3. Rheological Properties

**Figure 3:**
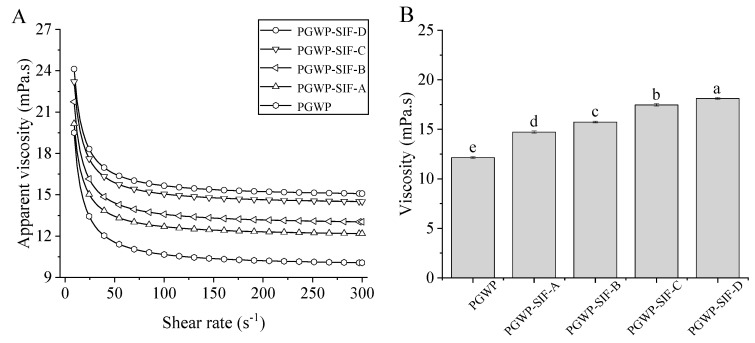
Effects of the SIF contents on flow behavior (**A**) and viscosity at 200 s^−1^ (**B**) of PGWP-SIF. Different subscript letters indicate a significant difference (*P* < 0.05). Error bars represent standard deviation of the means.

The rheological properties of PGWP and PGWP-SIF are shown in Figure 3. All samples showed shear-thinning behavior (Figure 3A). This may be due to that the interaction between particles were decreased at high shear rates, causing a decrease in the size of dispersions, and thus leading to a decrease in the viscosity [26]. All PGWP-SIF showed significantly higher (*P* < 0.05) viscosity at 200 s^−1^ compared with the PGWP (Figure 3B). The increase in viscosity of the nanoparticles can be attributed to the entrapment of SIF into the PGWP networks. Values of viscosity at 200 s^−1^ for PGWP-SIF were increased (*P* < 0.05) as SIF content increased from 2.1 mg/mL to 3.0 mg/mL.

### 3.4. FT-IR Spectra

**Figure 4:**
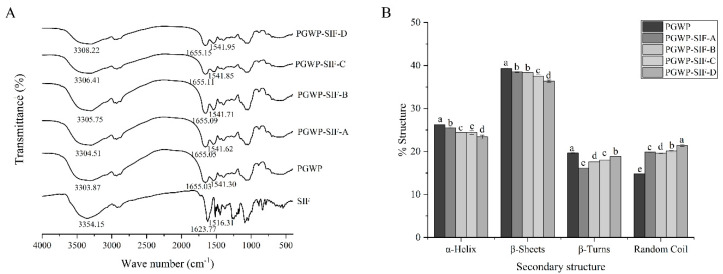
FT-IR spectra of SIF, PGWP, and PGWP-SIF (**A**) and the contents of secondary structures of PGWP and PGWP-SIF (**B**). Different subscript letters indicate a significant difference (*P* < 0.05). Error bars represent standard deviation of the means.

FT-IR analysis was used to determine the structural changes of PGWP when interacted with SIF. The amide I band 1600–1700 cm^−1^ (C=O stretch), amide II bands 1500–1600 cm^−1^ (C-N stretching combined with N-H bending modes), and amide A band at 3300 cm^−1^ (N-H stretching and hydrogen bonds) were used to explore the changes of the secondary structure of goat milk whey protein in PGWP-SIF [27]. FT-IR spectra of all samples were shown in Figure 4A. FT-IR spectra of the SIF showed characteristic peaks at 1623.77, 1516.31, and 3354.15 cm^−1^. The PGWP showed bands at 1655.03, 1541.30, and 3303.87 cm^−1^, which may belong to amide I, amide II, and amide A. After interacting with SIF, a hypsochromic shift occurred for the absorption band of PGWP-SIF. The peak in PGWP at 1655.03 cm^−1^ was shifted to 1655.15 cm^−1^ for PGWP-SIF, indicating that the addition of SIF affected the formation of the PGWP-SIF through the interaction related to C=O between PGWP and SIF. The peak at 1541.30 cm^−1^ (PGWP) shifted to 1541.95 cm^−1^ (PGWP-SIF) and may be due to the interactions between PGWP and SIF through C-N and N-H groups. The peak at 3303.87 cm^−1^ (PGWP) shifted to 3308.22 cm^−1^ (PGWP-SIF) and indicated a formation of hydrogen bonds between PGWP and SIF, suggesting that phenolic hydroxyl groups were involved in the non-covalent interaction between PGWP and SIF.

Contents of the secondary structure of proteins were calculated by using Amide I band. The results are shown in Figure 4B. When compared with PGWP, content of α-helix and β-sheet in PGWP-SIF decreased from 26.20 ± 0.07% to 23.39 ± 0.42%, and 39.32 ± 0.06% to 36.36 ± 0.25%, respectively. Results indicated that secondary structures of goat milk whey protein were transformed from ordered to disordered when combined with SIF.

### 3.5. Fluorescence Spectra

#### 3.5.1. Inherent Fluorescence

**Figure 5:**
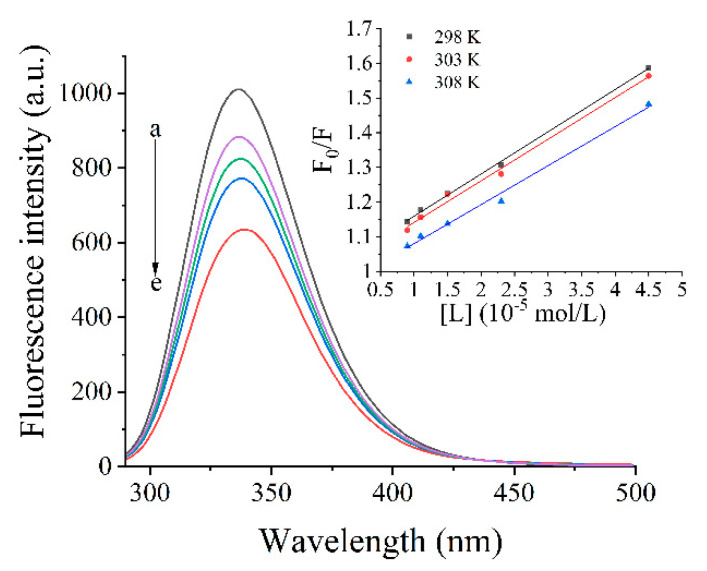
Fluorescence emission spectra of PGWP-SIF systems in 10 mM PB pH 7.4. a–e, PGWP, PGWP-SIF-A, PGWP-SIF-B, PGWP-SIF-C, PGWP-SIF-D. The Stern–Volmer plots for the quenching of PGWP by SIF at different temperatures.

Fluorescence intensity of PGWP and PGWP-SIF are shown in Figure 5. The PGWP-SIF showed lower fluorescence intensity than PGWP, indicating that SIF quenched the intrinsic fluorescence of PGWP. The fluorescence intensity decreased as SIF contents increased. Additionally, in the presence of SIF, the maximum peak position slightly shifted to a larger wavelength from 335 nm to 339 nm, which indicated that more fluorophores were exposed to the solvent, and thus implying a change in the polarity of tryptophan residues. The results suggested that some hydrophobic residues were buried during the interaction of SIF with PGWP, leading to the changes of structure in PGWP, and eventually improving the hydrophilicity of the medium.

#### 3.5.2. Stern–Volmer Analysis of Quenching Data

To further understand the interactions between PGWP and SIF, the Stem-Volmer equation was used to analyze the data at temperatures of 298, 303, and 308 K, as follows in Equation (2):.

F_0_/F = 1 + Ksv [L] = 1 + Kq τ_0_ [L] (2)

where F_0_ and F are fluorescence intensities of PGWP and PGWP-SIF, respectively; [L] is the concentration of SIF; Ksv is the Stern–Volmer quenching constant; Kq is the biological macromolecules quenching constant; and τ_0_ is the average lifetime of the biomolecule without quencher (τ_0_ = 10^−8^ s) [28].

It was reported that Kq larger than 2.0 × 10^10^ L/mol/s indicated static quenching interaction, while q smaller than 2.0 × 10^10^ L/mol/s indicated dynamic quenching interaction. Additionally, for static quenching, higher temperature indicated lower quenching constant, while dynamic quenching had the opposite trend [29]. From Table 1, it can be seen that values of Kq were greater than 2.0 × 10^10^ L/mol/s, and the Ksv decreased at higher temperatures, which suggested that the interaction was static quenching.

For static quenching, the binding constant (Ka) and the number of binding sites (n) conformed to the Equation (3) [30]. The slope and the intercept values of a plot of log [(F_0_ − F)/F] versus log [L] give n and Ka values, respectively.

log ((F_0_ − F)/F) = log Ka + n log [L] (3)

Values of n and Ka parameters are shown in Table 1. Values of n were close to 1, indicating that PGWP had only one binding site for SIF. On the other hand, values of Ka increased as temperature increased, indicating endothermic binding reaction. The result indicated that the stability of PGWP-SIF increased as temperature increased. Our results were similar with findings of Jia et al. [19], who reported that the interaction of β-lactoglobulin with epigallocatechin-3-gallate was endothermic and the stability increased as temperature increased.

#### 3.5.3. Thermodynamic Parameters

The four main interaction forces between polyphenols and proteins are hydrogen bonds, electrostatic forces, hydrophobic forces, and van der Waals forces [31]. The main interaction forces can be obtained by calculating the thermodynamic parameters using van’t Hoff Equations (4) and (5), as follows:

ln Ka = −ΔH/RT + ΔS/R(4)

ΔG = ΔH − TΔS(5)

where ΔH is the enthalpy change, ΔS is the entropy change, ΔG is free energy change, R is the gas constant (8.314 J/mol/K), T is the reaction temperature, and Ka is the binding constant at the temperatures of 298, 303, and 308 K. ΔH, ΔS, and ΔG could be acquired by Equations (3) and (4) [32].

Ross and Subramanian [33] reported that ΔS and ΔH > 0 indicated hydrophobic forces, ΔS and ΔH < 0 indicated van der Waals forces, and ΔH < 0 and ΔS > 0 indicated electrostatic forces. From Table 1, it can be seen that ΔG < 0, indicating that the interaction between PGWP and SIF was spontaneous [34]. Both ΔH and ΔS values > 0, indicating that hydrophobic interaction was involved in the interaction between PGWP and SIF. This was similar to the findings of Xu et al. [35], who reported that the interaction between β-lactoglobulin and theaflavin/chlorogenic acid/delphinidin-3-O-glucoside was mainly maintained by hydrophobic forces.

#### 3.5.4. Synchronous Fluorescence Spectra

**Figure 6:**
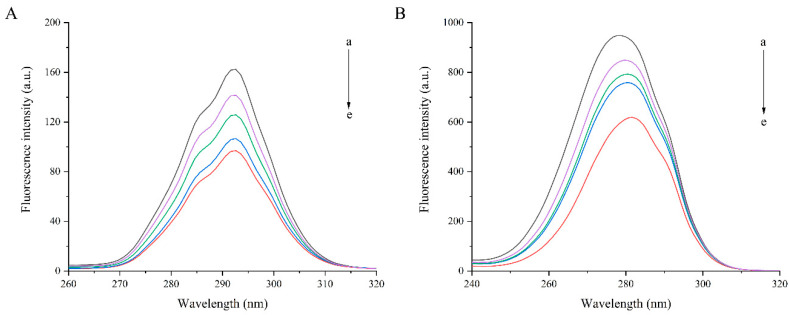
Synchronous fluorescence spectra of PGWP-SIF systems, a–e, PGWP, PGWP-SIF-A, PGWP-SIF-B, PGWP-SIF-C, PGWP-SIF-D. (**A**: Δλ = 15 nm, **B**: Δλ = 60 nm).

Synchronous fluorescence analysis was used to study effects of SIF on structure of PGWP and obtain information about tyrosine residues and tryptophan residues at synchronous spectrum performed with Δλ at 15 or 60 nm, respectively [19,35]. Some doubts about the effectiveness of the method were recently reported by Bobone et al. [36]. From Figure 6, it can be seen that as SIF content increased, the fluorescence intensity decreased in both fluorescence spectra, which indicated that the binding of SIF with PGWP exposed more chromophores into the solvent and led to a decrease in the fluorescence intensity.

### 3.6. Differential Scanning Calorimetry (DSC)

**Figure 7:**
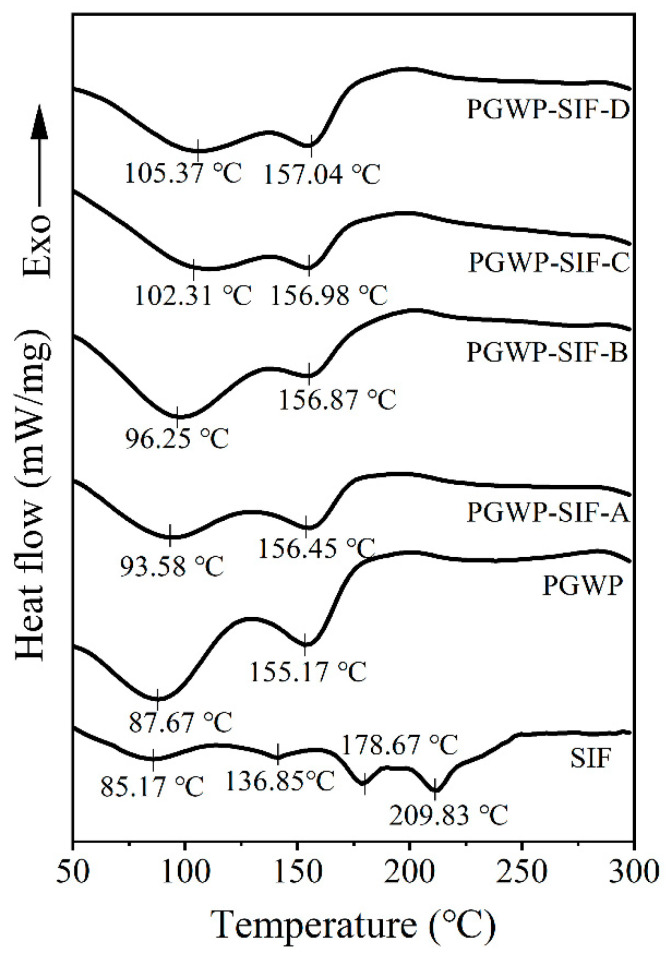
The differential scanning calorimeter (DSC) curves of SIF, PGWP, and PGWP-SIF.

DSC analysis was used to investigate the thermal property of nanoparticles. DSC curves of all samples were shown in Figure 7. The pure SIF showed four endothermic peaks at approximately 85.17, 136.85, 178.67, and 209.83 °C. The first three peaks may be attributed to three molecular forms in SIF, including genistein, daidzein, and glycitein [4]. The PGWP exhibited two endothermic peaks at 87.67 °C and 155.17 °C, and the PGWP-SIF showed two endothermic peaks at 105.37 °C and 157.04 °C. The result indicated that the thermal stability of the PGWP-SIF improved. Moreover, the characteristic peaks of SIF disappeared in PGWP-SIF, which may be because SIF were encapsulated into PGWP microspheres. Yang et al. [27] also reported that the disappearance of endothermic peaks of pyrogallic acid in the nanoparticle was due to the covalent interactions between pumpkin seed protein isolate and pyrogallic acid.

### 3.7. Microstructure

**Figure 8:**
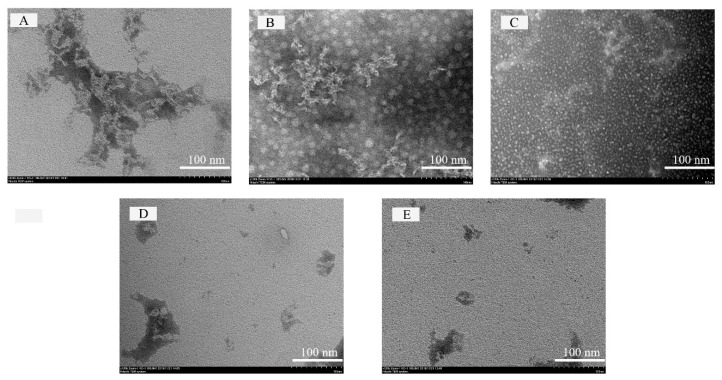
Transmission electron microscopy micrographs of PGWP and PGWP-SIF. PGWP (**A**); PGWP-SIF-A (**B**); PGWP-SIF-B (**C**); PGWP-SIF-C (**D**), and PGWP-SIF-D (**E**).

Images of PGWP and PGWP-SIF obtained by transmission electron microscope (TEM) are presented in Figure 8. The morphology of the PGWP-SIF was related to the SIF contents. At the low SIF contents (Figure 8B,C), the microspheres were homogeneously spherical in shape with smaller particle size, suggesting that the nanoparticles were homogeneously dispersed. At the large SIF contents (Figure 8D,E), irregularly larger-sized particles were formed, and this may be due to the formation of inter-surface networks between the PGWP-SIF and unencapsulated SIF. The microstructure was consistent with the mean particle size obtained from dynamic light scattering data (Figure 2A).

## 4. Discussion

Numerous studies have reported the beneficial properties of soy isoflavones (SIF). However, its application in the food industry and pharmaceuticals are limited owing to its low solubility in water and poor bioavailability. Goat milk whey protein has excellent emulsifying and foaming properties [6,7], which can be used to produce bioactive compounds-loaded whey protein to expand their applications in the production of functional food products as well as for designing new drug delivery systems [37]. In this study, we prepared SIF nanoparticles using polymerized goat milk whey protein (PGWP) as wall material.

The encapsulation efficiency is the index used to indicate how many SIF were loaded in the nanoparticles. The encapsulation efficiencies of SIF in the nanoparticles were higher than that obtained from gel beads of soybean hull polysaccharides by Wang et al. [5], which may be attributed to the differences in methods and conditions, and the result indicated that it was feasible to encapsulate SIF using PGWP as wall material. Particle size and zeta potential are important properties to provide valuable information on micro-encapsulated compounds regarding the formation of stable formulations. The particle size of the nanoparticles ranged from 135 nm to 155 nm due to the concentrations of SIF in PGWP-SIF. The values were similar to previous report by Khan et al. [11], who prepared whey protein isolate-DIM (3,3′-Diindolylmethane) nanoparticles to a mean particle size of 96–157 nm. In the study, concomitant observations were obtained from transmission electron microscope (TEM) images and dynamic light scattering (DLS) analyses, the microspheres were homogeneously spherical in shape. However, compared with the dynamic light scattering data, the diameter of particles appeared smaller in the TEM images, which may be due to the different principles of the two analytical methods. In addition, all the nanoparticles had values of zeta potential below −30 mV, which suggested that the samples seemed to be stabilized [24]. The nanoparticles with higher surface charge aggregated less, which suggested that they were somewhat more stable. These results suggested that PGWP could be considered as a promising encapsulation agent for the incorporation of bioactive compounds such as SIF.

Fourier transform infrared spectroscopy (FT-IR) analysis can be used to investigate the potential interactions between SIF and PGWP. FT-IR spectra of the SIF showed characteristic peaks were related to the stretching vibration of the aromatic ring and aromatic ketone [5]. The characteristic absorption peaks of SIF in nanoparticles spectrum increased from 1623.77 cm^−1^ and 1516.31 cm^−1^ to 1655.15 cm^−1^ and 1541.95 cm^−1^, respectively, which suggested that the successful formation of PGWP-SIF nanoparticles. The results were consistent with the report described by Wang et al. [38], who investigated the formation of complexes between spiral dextrin sub-fraction and soy isoflavones. After interacting with SIF, the amide I band of PGWP changed, which may be due to hydrophobic interactions between the aromatic ring of SIF and the hydrophobic amino acids of proteins [39]. The amide I band was the most useful for infrared spectroscopic analysis of the secondary structure of proteins [40]. From Figure 4B, it could be observed that the α-helix and β-sheet contents for PGWP decreased after adding SIF, suggesting that the interactions between PGWP and SIF could lead to the alteration of PGWP secondary structure. It is presumable that SIF were non-covalently grafted onto PGWP, resulting in the partly unfolded protein, which may lead to the exposure of buried amino acids and promote hydrophobic interactions [41]. These results indicated that the secondary structure of goat milk whey protein changed after interacting with SIF, and the interaction between PGWP and SIF was probably through hydrogen bonds or hydrophobic interactions.

To confirm that the conformation of PGWP was changed after interacting with SIF, we studied the fluorescence spectrum. Fluorescence spectrum was widely used to study mechanisms of the interactions between proteins and small molecules and the microenvironment changes of proteins. The main components of whey proteins are β-lactoglobulin (β-LG) and α-lactalbumin (α-LA). Each β-LG molecule has two tryptophan residues and four tyrosine residues, while four tryptophan residues are found in α-LA molecule [42]. The fluorescence of tyrosine and tryptophan was excited by different wavelengths to analyze the structural changes of proteins. Therefore, the inherent fluorescence is a useful approach to study the structural transition and binding properties of protein. Fluorescence experiments proved that SIF quenched PGWP fluorescence strongly in static mode. The nature of the interaction forces between SIF and PGWP can be partially unveiled by studying the thermodynamic parameters of the system, and the results suggested that the main interaction force was hydrophobic interaction [33]. Synchronous fluorescence confirmed that SIF affected the conformation of PGWP by interacting with its tyrosine and tryptophan residues. In addition, tryptophan residues had a stronger fluorescence quenching effect than that of tyrosine residues, which indicated that tryptophan residues played an important role in fluorescence quenching. Combining the fluorescence spectrum and the synchronous fluorescence spectrum, the amino acids involved in the reaction were tryptophan and tyrosine, and it can be inferred that tryptophan and tyrosine residues were involved in the hydrophobic interaction. To further confirm that SIF were encapsulated inside the nanoparticles, we studied the thermal properties of SIF before and after encapsulation. The characteristic peaks of SIF disappeared in that of PGWP-SIF, which may be attributed to that SIF were entrapped in PGWP nanoparticles. These results were in agreement with the report of Wang et al. [38], who observed that the disappearance of the endotherm of soy isoflavones at 184.54 °C in the thermogram of the complexes may be due to the formation of spiral dextrin sub-fraction/soy isoflavones complexes. This work provided some comprehensive understanding about the interactions between SIF and PGWP, and these characteristics made PGWP a novel wall material for the encapsulation of SIF. However, this research has some limitations, the antioxidant activity and in vitro release behavior of the nanoparticles will be carried out in the next study, and molecular modeling study will be performed to predict the precise binding sites of soy isoflavones in goat milk whey protein. Our study indicated that the polymerized goat milk whey protein can be considered as a promising carrier for encapsulating bioactive compounds. The results may be helpful in expanding the industrial application of soy isoflavones in functional foods. The study laid the foundation for further research into the interaction between goat milk whey protein and soy isoflavones.

## 5. Conclusions

Soy isoflavones nanoparticles using polymerized goat milk whey protein as wall material were prepared and characterized in this study. The results suggested that polymerized goat milk whey protein prepared directly from milk was suitable to encapsulate soy isoflavones with high encapsulation efficiency, and hydrophobic interaction was considered to be the main force in the formation of the nanoparticles. The findings will be helpful for the use of polymerized goat milk whey protein as a carrier material for hydrophobic bioactive compounds.

## Figures and Tables

**Table 1:** Relevant parameters of the Stern–Volmer quenching constant (Ksv), the biological macromolecules quenching constant (Kq), the binding constant (Ka), and the number of binding sites (n) were calculated from Stern–Volmer and double log plots at different temperatures. Thermodynamic parameters of enthalpy (ΔH), entropy (ΔS) and free energy (ΔG) changed based on the van’t Hoff equation.

T (K)	Ksv (10^4^ L/mol)	Kq (10^12^ L/mol)	R^2^	Ka (10^5^ L/mol)	n	R^2^	ΔH (KJ/mol)	ΔG (KJ/mol)	ΔS (J/mol.K)
298	1.22 ± 0.1	1.22 ± 0.1	0.9988	7.80 ± 0.1	1.13 ± 0.02	0.9969	7.81 ± 0.1	–33.74 ± 0.1	139.42 ± 1.2
303	1.20 ± 0.2	1.20 ± 0.2	0.9932	8.22 ± 0.1	0.93 ± 0.01	0.9873		–34.43 ± 0.2	
308	1.13 ± 0.2	1.13 ± 0.2	0.9927	8.34 ± 0.1	0.86 ± 0.02	0.9929		–35.13 ± 0.2	
